# Regional factors rather than forest type drive the community structure of soil living oribatid mites (Acari, Oribatida)

**DOI:** 10.1007/s10493-012-9546-9

**Published:** 2012-03-30

**Authors:** Georgia Erdmann, Stefan Scheu, Mark Maraun

**Affiliations:** J.F. Blumenbach Institute of Zoology and Anthropology, Georg August University Göttingen, Berliner Str. 28, 37073 Göttingen, Germany

**Keywords:** Temperate forests, Management, Natural forest, Oribatid mites, Density, Diversity, Community structure

## Abstract

Most European forests are managed by humans. However, the manner and intensity of management vary. While the effect of forest management on above-ground communities has been investigated in detail, effects on the below-ground fauna remain poorly understood. Oribatid mites are abundant microarthropods in forest soil and important decomposers in terrestrial ecosystems. Here, we investigated the effect of four forest types (i.e., managed coniferous forests; 30 and 70 years old managed beech forests; natural beech forests) on the density, diversity and community structure of oribatid mites (Acari). The study was replicated at three regions in Germany: the Swabian Alb, the Hainich and the Schorfheide. To relate changes in oribatid mite community structure to environmental factors, litter mass, pH, C and N content of litter, fine roots and C content of soil were measured. Density of oribatid mites was highest in the coniferous forests and decreased in the order 30 years old, 70 years old, and natural beech forests. Mass of the litter layer and density of oribatid mites were strongly correlated indicating that the litter layer is an important factor regulating oribatid mite densities. Diversity of oribatid mites was little affected by forest type indicating that they harbor similar numbers of niches. Species composition differed between the forest types, suggesting different types of niches. The community structure of oribatid mites differed more strongly between the three regions than between the forest types indicating that regional factors are more important than effects associated with forest type.

## Introduction

Central Europe comprises old cultivated land, modified by humans since thousands of years. Different management regimes influenced today’s Fauna and Flora. Today about 30 % of Central Europe is covered by forests (European Environment Agency 2005). The overwhelming part of forest areas in Europe is subject to silviculture. The major form of forest management type is age-class forest (Fischer et al. 2010). These forests lack variability in age structure and stand composition. For economic reasons managed forests are dominated by beech, oak, pine and spruce (Ellenberg 1996). Beech is the natural occurring and most common tree species in central Europe, in contrast to spruce which was introduced into lowland ecosystems. In dry lowland regions pine was planted instead of spruce (Ellenberg 1996).

Silvicultural practices alter natural succession processes and decrease spatial heterogeneity of resources and environmental conditions (Halpern and Spies 1995). Thinning, harvesting and hauling of mature trees take place in managed forests causing drastic disturbances. The type of forest management affects plant diversity (Halpern and Spiess 1995) and community structure of above ground animals, such as carabid beetles (Werner and Raffa 2000; Niemelä et al. 2007) and spiders (Brennan et al. 2006). Furthermore, ant, carabid beetle and spider diversity increase with increasing forest age (Niemelä et al. 1996). Paillet et al. (2010) found an overall increase in species richness in natural temperate and boreal forests in Europe in comparison to managed forests with bryophytes, lichens, fungi, saproxylic beetles and carabids being most affected. However, the effect of different forest management types on below ground organisms has been little studied (but see e.g. Lindo and Visser 2004; Cassagne et al. 2006). Forest management may reduce soil organic matter, increase soil compaction, change plant cover and modify microclimate. All of these effects affect the distribution, composition and activity of soil animal communities (Marshall 2000).

Despite the seemingly homogeneous habitat soil organisms live in (Giller 1996), the diversity of soil animals is very high. This phenomenon has been termed the “enigma of soil animal diversity” (Anderson 1975). Especially the microarthropod taxa, such as Collembola and oribatid mites (Acari), have a high diversity with about 100 species in European forests (Norton and Behan-Pelletier 2009). Moreover, oribatid mites are abundant in virtually any forest reaching densities between 20,000 ind./m² in base rich forests and 200,000 ind./m² in acidic forests (Maraun and Scheu 2000). Oribatid mites are mainly decomposers feeding on dead organic material and fungi; however, recently it has been postulated that they also live directly or indirectly from rhizosphere carbon (Pollierer et al. 2007), feed on lichens (Erdmann et al. 2007) as well as on dead or living animals (Maraun et al. 2011; Heidemann et al. 2011). Stable isotope signatures indicate that they span about 3–4 trophic levels including decomposers, fungal feeders, scavengers and predators (Schneider et al. 2004).

The aim of this study was to identify general patterns of the effect of forest management and forest type on density, diversity and community structure of soil living oribatid mites in four forest types: 30 and 70 years old beech forests *(Fagus sylvatica)*, natural beech forests (mature trees being approx. 120 years old) and 70 years old coniferous forests at large geographic ranges in Central Europe. The study was replicated at three regions in Germany; the Swabian Alb (Baden-Wuerttemberg), the Hainich (Thuringia) and the Schorfheide (Brandenburg) spanning a latitudinal gradient of more than 500 km. Due to soil and climate conditions coniferous forests in the Hainich and Alb consist of spruce (*Picea abies*) and in Schorfheide of pine (*Pinus sylvestris*).

We hypothesized that (1) oribatid mite density is highest in forests with thick organic layers (i.e., in acidic coniferous forests) providing habitat and food; (2) the diversity of oribatid mites is highest in unmanaged (natural) forests due to low disturbance and high microhabitat diversity and heterogeneity; and (3) the community structure of oribatid mites differs between forest types providing different types of niches.

## Materials and methods

### Study sites

We used experimental forest sites at three regions in Germany selected for long-term monitoring in the framework of the Biodiversity Exploratories, an integrative ecosystem research project (http://www.biodiversity-exploraties.de). The regions are located in the Swabian Alb, a low-mountain range in South-Western Germany (460–860 m a.s.l.), the Hainich, a hilly region in central Germany (285–550 m a.s.l.), and the Schorfheide, a glacial formed landscape in North-Eastern Germany (3–140 m a.s.l.). Parent rock is Jurassic shell limestone in the Swabian Alb, Triassic limestone in the Hainich and glacial till in the Schorfheide. Soil types at the study sites in the Swabian Alb were mainly Cambisols and partially Leptosols. In the Hainich Luvisol represented the main soil type and to a lesser extent Cambisol and Stagnosol. The Schorfheide is dominated by Cambisols interspersed with Luvisols (Fischer et al. 2010). Acidity of the soil ranged from pH 3.3 ± 0.19 in the Schorfheide to 4.51 ± 0.72 in the Swabian Alb to 4.59 ± 0.67 in the Hainich. The mean annual precipitation in the Swabian Alb is 700–1,000 mm with a mean annual temperature of 6–7 °C; respective values at the Hainich and the Schorfheide are 500–800 mm and 6.5–8 °C, and 500–600 mm and 8–8.5 °C. More details on the Biodiversity Exploratories are given in Fischer et al. (2010).

### Sampling design

Four forest types were investigated in each of the three regions, including approximately 70 year old coniferous forests (*P. abies* in the Swabian Alb and Hainich; *P. sylvestris* in the Schorfheide), 30 and 70 years old beech forests (*F. sylvatica*) and natural beech forests with mature trees being 120–150 years old. The coniferous forests as well as the 30 and 70 years old beech forests were planted forests (=age class forests); the natural forests were taken out of management at different dates (for details see Fischer et al. 2010).

Soil samples were taken in April and May 2008. The four forest types were replicated four times in each of the three regions resulting in 48 sampled forests. The forests were at least 1 km aside from each other. In each forest two soil cores were taken from a 5 × 5 m square and pooled for statistical analysis. Soil cores were taken with a soil corer (5 cm Ø) and separated in organic and soil layer (4 cm thickness). Soil animals were extracted by heat (Macfadyen 1961) from both layers separately but data were pooled for the statistical analyses. Until determination animals were stored in 70 % ethanol. Adult oribatid mites were determined using Weigmann (2006). Suctobelbidae and Brachychthoniidae were determined to family-level; juvenile oribatid mites were counted.

### Environmental factors

The litter layer of the soil cores used for arthropod extraction was weighed after drying during heat extraction. Soil pH of the soil cores was measured in 0.01 M CaCl_2_ solution. Carbon and nitrogen content of the litter and of fine roots, taken out of the soil cores after heat extraction, as well as carbon content of the soil were measured with an elemental analyzer (NA 1500, Carlo Erba, Milan).

### Statistical analysis

Data of oribatid mite density and diversity were log transformed (counted data) or arc-sin transformed (proportional data) to improve homogeneity of variances and normal distribution; and analyzed using two-factorial ANOVA with the factors region (Swabian Alb, Hainich, Schorfheide) and forest type (coniferous forest, beech 30 years old, beech 70 years old, natural beech forest). In case of significance post hoc tests (Tukey’s HSD) were performed to inspect differences between means. Means and standard deviations given in text and figures are based on untransformed data. Correlations of oribatid mite density with environmental factors were tested using Pearson Correlation. ANOVA and correlations were calculated with STATISTICA 9.1 software package (Statsoft Tulsa, USA).

The impacts of different levels of the treatments forest type and region on oribatid mite community composition were analyzed separately conducting two Discriminant Functional Analyses (DFA) following the procedure given in Tiunov and Scheu (2000). Species which occurred in less than four plots were excluded from the analysis. The DFA was performed using STATISTICA 9.1 software package (Statsoft Tulsa, USA). Multidimensional scaling (MDS) was carried out prior to DFA to reduce the number of dimensions. Reduction to six dimensions was the best solution to minimize dimensions. Forest type or region were used as grouping variables in DFA and Squared Mahalanobis distances between group centroids and the reliability of the sample classifications were determined. The extracted significant roots were correlated (Pearson) with environmental data to identify factors which are responsible for differences among forest types and regions.

Ordination techniques were applied to correlate species and environmental factors structuring the communities in the regions and forest types. For Canonical Correspondence Analysis (CCA) species (present in at least 4 independent samples) and environmental data were log-transformed; forest types were coded as supplementary variables. CCA was performed using CANOCO 4.5 (Jongman et al. 1995; ter Braak and Šmilauer 2002).

## Results

### Oribatid mite density

**Fig. 1:**
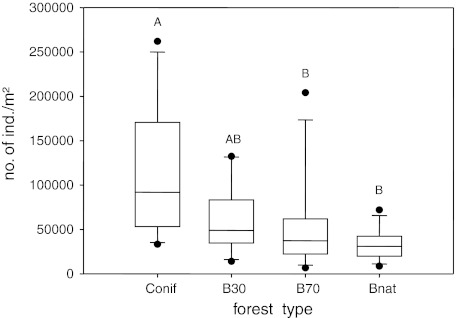
Oribatid mite density in different forest types (*Conif* coniferous forest, *B30* 30 year old beech forest, *B70* 70 year old beech forest, *Bnat* natural beech forest). *Boxes*’ indicate the 25th and 75th percentile, the *line* in the *box* marks the median, *whiskers* map the 90th and 10th percentile; *dots* display outliers; *different letters* indicate significant differences between means (Tukey’s honestly significant difference test, *p* < 0.05)

Oribatid mite density (adults and juveniles together) differed significantly among the four forest types (F_3,36_ = 7.36; *p* < 0.001; Fig. 1), it decreased from coniferous forests (117.352 ± 74.266 ind./m²) over 30 years old beech forests (59.920 ± 38.721 ind./m²) and 70 years old beech forests (53.683 ± 53.953 ind./m²) to natural beech forests (32.985 ± 17.134 ind./m²). Oribatid mite densities did not differ significantly among the three regions (F_2,36_ = 2.61; *p* = 0.09; 59.814 ± 45.126, 53.451 ± 61.725 and 84.690 ± 64.799 ind./m² for the Swabian Alb, Hainich and Schorfheide, respectively).

The proportion of juveniles (approx. 25 % of all individuals) did not differ significantly among forest types (F_3,36_ = 1.08; *p* = 0.37) but among the regions (F_2,36_ = 6.49; *p* = 0.004). The proportion of juveniles was at a maximum in the Schorfheide with 32.5 % ± 13 and significantly lower in the Swabian Alb with 19.3 % ± 10.4, with the proportion in the Hainich being intermediate 23.1 % ± 9.4 (and not significantly different from the two other regions).

Oribatid mite density was positively correlated with the mass of litter layer (r² = 0.18, *p* = 0.003), the concentration of carbon in litter (r² = 0.12, p = 0.015) and the concentration of carbon in soil (r² = 0.10, *p* = 0.026), and negatively correlated with pH (r² = 0.23, *p* = 0.001), concentration of nitrogen in fine roots (r² = 0.14, *p* = 0.01) and concentration of nitrogen in litter (r² = 0.11, *p* = 0.03).

### Species numbers of oribatid mites

**Fig. 2:**
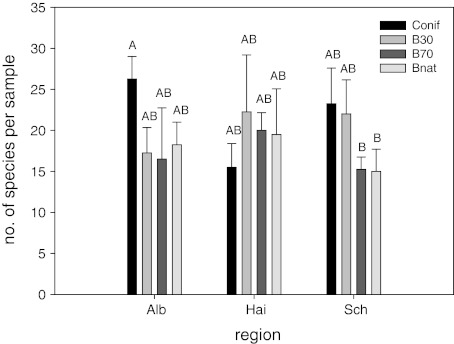
Number of oribatid mite species per sample in the three regions of the Biodiversity Exploratories (*Alb* Swabian Alb, *Hai* Hainich, *Sch* Schorfheide) and the forest types (*Conif* coniferous forest, *B30* 30 year old beech forest, *B70* 70 year old beech forest, *Bnat* natural beech forest; *different letters* indicate significant differences between means [Tukey’s honestly significant difference test, *p* < 0.05)]

Overall, 114 species of oribatid mites were found. The total number of species was highest in the Swabian Alb (78), lower in the Schorfheide (65), and lowest in the Hainich (57), with on average 79 and 78 species in coniferous and 30 years old beech forests, respectively, and 64 species in both the 70 years old and natural beech forests. The average number of species per soil sample differed significantly among forest types (F_3,36_ = 3.02, *p* = 0.04). Although the total number of oribatid mites was highest in the Swabian Alb, lower in the Schorfheide and lowest in the Hainich, the average number of oribatid mite species per sample did not differ among regions (F_2,36_ = 0.08, *p* = 0.92), but variations with forest type differed among regions (significant region × forest type interaction; F_6,36_ = 3.77, *p* = 0.005), with the number of oribatid mite species per sample being highest in the coniferous forests of the Swabian Alb and lowest in the 70 years old and natural beech forests of the Schorfheide (Fig. 2).

### Community structure of oribatid mites

Oribatid mite communities differed significantly among the four forest types (DFA: Wilk’s Lambda = 0.21, F_18,110_ = 4.57, *p* < 0.0001). The first of three extracted roots was significant and the oribatid mite communities of the forest types were separated along this axis. Oribatid mite communities of coniferous forests differed significantly from beech forests; but also among beech forests they differed significantly between 30 years old forests and natural forests. Generally, oribatid mite communities changed gradually from coniferous forests over 30 years old beech forests and 70 years old beech forests to natural beech forests. The mass of litter layer correlated positively (r² = 0.21, *p* = 0.001) and nitrogen concentrations in fine roots correlated negatively (r² = 0.118, *p* = 0.017) with the first DFA root.

Oribatid mite species compositions also significantly differed among the three regions (DFA: Wilk’s Lambda = 0.02, F_12,80_ = 32.63, *p* < 0.0001). The oribatid mite community of the Schorfheide was separated along the first root from the other regions (Mahalanobis Distance (MD) between Schorfheide and Swabian Alb 41.37, F_6,40_ = 49.03, *p* < 0.0001, and between Schorfheide and Hainich 32.97, F_6,40_ = 39.08, *p* < 0.0001). The second root separated Hainich and Swabian Alb (MD = 17.06, F_6,40_ = 20.22, *p* < 0.0001). Soil pH correlated negatively with the first root (r² = 0.524, *p* < 0.001) and positively with carbon concentrations in fine roots (r² = 0.205; *p* = 0.001). The second root correlated negatively with mass of litter layer (r² = 0.184, *p* = 0.002) and positively with nitrogen concentrations in fine roots (r² = 0.106, *p* = 0.024).

**Fig. 3:**
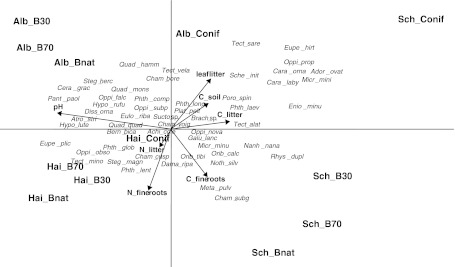
Canonical Correspondence Analysis (CCA) of oribatid mite species (in *italics*, for full species names see Appendix) at different forest types (*Conif* coniferous forest, *B30* 30 year old beech forest, *B70* 70 year old beech forest, *Bnat* natural beech forest) in the three regions of the Biodiversity Exploratories (*Alb* Swabian Alb, *Hai* Hainich, *Sch* Schorfheide) and the measured environmental factors (in *bold*; leaf litter = mass of litter layer; C and N of litter, soil or fine roots = concentrations of C or N in leaf litter, mineral soil or fine roots); Eigenvalues of the first and second axis: 0.234 and 0.132, respectively

Oribatid mite community varied most among the three regions but not among forest types (Canonical Correspondence Analysis, CCA; Fig. 3). Along the first CCA axis Schorfheide was separated from Swabian Alb and Hainich. Among environmental factors soil pH correlated closest with the first axis with higher acidity in the Schorfheide and more alkaline conditions in the Swabian Alb and Hainich. Oribatid mite communities of the Swabian Alb and Hainich also differed and were separated along the second axis. The second axis correlated closely with mass of litter layer with higher values in the Swabian Alb than the Hainich than in the Schorfheide, and with concentrations of carbon and nitrogen in fine roots with higher values in the Hainich than in the Swabian Alb. Some oribatid mite species analyzed in the CCA exclusively occurred in one of the three Biodiversity Exploratories: *Chamobates subglobulus*, *Microtritia minima* and *Oppiella propinqua* only occurred in the Schorfheide, *Oppiella obsoleta* and *Tectocepheus minor* only in the Hainich, and *Quadroppia hammerae* and *Tectocepheus velatus velatus* only in the Swabian Alb.

In each of the three regions oribatid mite community composition in coniferous forests differed from that in beech forests. CCA indicated that this is due to lower pH in soil and higher mass of litter layer in coniferous forests. Coniferous forests were characterized by the oribatid mite species *Chamobates borealis*, *Phthiracarus longulus*, *Platynothrus peltifer*, *Tectocepheus velatus alatus* (except in the Swabian Alb) and high densities of Brachychthoniidae and Suctobelbidae. Oribatid mite species typical for 70 years old and natural beech forests were *Atropacarus striculus*, *Ceratozetes gracilis* (except in the Schorfheide), *Chamobates cuspidatus*, *Damaeus riparius* and *Steganacarus magnus*. The oribatid mite community in coniferous forests in Schorfheide (pine) differed from each of the other sites with high abundances of *Adoristes ovatus*, *Carabodes labyrinthicus*, *Carabodes ornatus*, *Oppiella propinqua* and *Tectocepheus velatus sarekensis*. Coniferous forests in Schorfheide were characterized by low soil pH, high mass of litter layer and low concentrations of nitrogen and carbon in fine roots.

## Discussion

### Oribatid mite density

Oribatid mite densities in the four forest types were highest in the coniferous forests (~120.000 ind./m²), lower in the 30 years old (~60.000 ind./m²) and the 70 years old (~50.000 ind./m²) beech forests and lowest in the natural beech forests (~30.000 ind./m²). High density of oribatid mites in coniferous compared to deciduous forests of the temperate zone have been reported before. Maraun and Scheu (2000) concluded that it is the humus form that affects oribatid mite densities with high densities in acidic mor and moder systems and low densities in base rich mull soils. A large number of biotic and abiotic factors correlate with the humus form including pH and presence of macroarthropods (Schaefer and Schauermann 1990), complicating disentangling causal mechanisms responsible for the differential density of oribatid mites. In our study oribatid mite density correlated closely with the mass of litter layer suggesting that the thickness of the litter layer rather than the humus form determines oribatid mite densities (Migge et al. 1998; Osler et al. 2006). Forest type and tree species affect litter thickness thereby also affecting oribatid mite density. Similar to the correlation between mass of litter layer and oribatid mite density, soil pH also correlated with oribatid mite density but the correlation was negative. Presumably, soil acidity does not directly affect oribatid mite densities but via controlling the density of macro-decomposers in particular earthworms. As documented previously (Maraun and Scheu 2000; Migge-Kleian et al. 2006) oribatid mites also reach high densities in base-rich soils not colonized by earthworms indicating that the presence of earthworms reduces the density of oribatid mites. This scenario suggests that via soil pH parent rock affects colonization of earthworms (and other macro-decomposers) which in turn affects the thickness of the litter layer which eventually influences the density of oribatid mites (and other mesofauna; cf. Eisenhauer 2010). The litter layer is essential for oribatid mites as it functions both as habitat and food resource (Ponge 1991; Schneider et al. 2004).

Oribatid mite densities were negatively correlated with the concentration of nitrogen in litter and fine roots. Again, this might be due to high densities of macro-decomposers at those nitrogen-rich sites. As parent rock, litter quality essentially drives the density of macro-decomposers including earthworms (Scheu et al. 2003; Salamon et al. 2005). Hence, by favouring macro-decomposers high quality litter may result in shallow litter layers; indeed, in the present study the mass of litter layer correlated negatively with concentrations of nitrogen in the litter and fine roots.

Oribatid mite densities were positively correlated with the concentration of carbon in soil. Apart from feeding on litter material and microorganisms living therein oribatid mites also acquire resources from the soil (Albers et al. 2006; Pollierer et al. 2007). A high proportion of carbon in soil, which may have originated from root exudates which in turn have supported the growth of fungi (Frey et al. 2003; Butenschoen et al. 2007; Broeckling et al. 2008), may have fostered fungal feeding oribatid mites.

Unexpectedly, oribatid mite densities did not differ significantly among the three regions (Swabian Alb, Hainich, Schorfheide). This indicates that regional factors, such as precipitation, altitude and temperature, which differ among the regions (Fischer et al. 2010) affect oribatid mite densities less than forest type.

The proportion of juvenile oribatid mites was highest in the Schorfheide. This is likely due to the high number of parthenogenetic species of the taxon Desmonomata (e.g., *Nothrus sylvestris* and *Nanhermannia nana*) in the Schorfheide. Those species develop slowly and have generation times of several years (Palmer and Norton 1991). Due to their slow development juvenile Desmonomata are present in high numbers throughout the year.

### Oribatid mite diversity

Oribatid mite diversity varied little with forest type and region. Only in the coniferous forests in the Swabian Alb oribatid mite diversity was somewhat higher than in the 70 years old and unmanaged beech forests of the Schorfheide. Previous studies also found oribatid mite diversity to be little affected by forest stand age (Zaitsev et al. 2002) or tree species (Migge et al. 1998; Sylvain and Buddle 2010). The somewhat higher diversity of oribatid mites in the coniferous forests at the Swabian Alb might be related to planting spruce trees on base rich Cambisols, which may have increased the number of niches. The low species number in the 70 years old unmanaged beech forests of the Schorfheide may at least in part result from the high density of Brachychthoniidae and Suctobelbidae which were not determined to species level.

The diversity of oribatid mites and factors affecting it are still little understood (Anderson 1978; Hansen 2000; Maraun et al. 2003). Results of the present study suggest that the number of (trophic) niches of oribatid mites in beech and coniferous forests in Central European forests is similar and little affected by forest management and forest type. The limited effect of forest management and forest type on soil living oribatid mite diversity contrasts strong effects on above ground organisms (Paillet et al. 2010). These differential effects deserve further attention.

### Oribatid mite community structure

Oribatid mite community composition differed between the coniferous and beech forests and between the young (30 years old) and the old (natural) beech forests. This suggests that the different forest types provide different niches for oribatid mites. Different niches are likely to be related to oribatid mite nutrition. It has been shown using a number of methods that oribatid mite species occupy very different trophic niches (Maraun et al. 2011; Schneider et al. 2004; Koukol et al. 2009; Heidemann et al. 2011).

Oribatid mite community structure in the beech forests changed gradually from the 30 years old over the 70 years old to the natural beech forests pointing to a slow but constant species turnover with ageing of the beech forests. This slow succession may be due to a parallel slow change in fungal and bacterial community structure (Visser 1995; Pennanen et al. 1999) but also in changes in the colonization by soil macrofauna (Scheu et al. 2003; Crow et al. 2009).

Oribatid mite communities differed significantly among each of the three Exploratories. Although oribatid mites are poor dispersers (Berthet 1964; Ojala and Huhta 2001; Lehmitz et al. 2011) dispersal limitation is unlikely to be responsible for these differences as most oribatid mite species are widespread and have a palaearctic or holarctic distribution (Weigmann 2006). The clear differences in oribatid mite community structure among the three regions point to the existence of different niches in the respective systems. Oribatid mite community structure can be predicted (Maraun and Scheu 2000) indicating that these communities do not assemble by chance which supports the importance of different niches for the community structure of oribatid mites. However, the factors responsible for these different oribatid mite communities in the respective forests are still little understood.

The oribatid mite communities of the four forest types within the investigated regions were more similar to each other than among regions. This points to the importance of regional factors, such as temperature, precipitation and parent rock, being more important as structuring forces for oribatid mite communities than forest types. Studying oribatid mite assemblages in forests of different harvesting regimes at local and regional scales Déchenê and Buddle (2009) also suggested regional factors to be superior to local factors.

As indicated by CCA soil acidity was the most important environmental factor for oribatid mite communities. However, soil acidity unlikely affects oribatid mite communities directly since most oribatid mite species tolerate even very acidic conditions (Hagvar 1990). Rather, as discussed above, soil pH affects colonization of the forests by soil macro-decomposers and this affects oribatid mites via changes in the thickness of the litter layer. Further, soil pH affects soil microorganisms (Baath and Anderson 2003; Dequiedt et al. 2011) which also is likely to affect oribatid mites.

### Conclusions

Overall, oribatid mite density varied significantly with forest type, whereas the diversity and community structure of oribatid mites was little affected. The most important factor for oribatid mite density was mass of litter layer being at a maximum in coniferous forests and at a minimum in old-growth natural beech forests. This indicates that forest types changing the thickness of the litter layer and strongly affect oribatid mite densities. Among abiotic factors, soil acidity strongly affects oribatid mite community structure but this likely is due to indirect effects via affecting macro-decomposers, in particular earthworms, which detrimentally affect oribatid mites via bioturbation i.e., by reducing the thickness of organic layers and by mixing litter and mineral soil. Generally, variations in oribatid mite community structure were more pronounced at the regional scale (among the three regions), than at the local scale among forest types (within the regions).

## Appendix

Achi_cole, *Achipteria coleoptrata* (Linné, 1758); Ador_ovat, *Adoristes ovatus* (C.L. Koch, 1839); Atro_stri, *Atropacarus striculus* (C.L. Koch, 1835); Bern_bica, *Berniniella bicarinata* (Paoli, 1908); Brach_sp., Brachychthoniidae; Cara_laby, *Carabodes labyrinthicus* (Michael, 1879); Cara_orna, *Carabodes ornatus*, Storkan, 1925; Cera_grac, *Ceratozetes gracilis* (Michael, 1884); Cham_bore, *Chamobates borealis* (Trägardh, 1902); Cham_cusp, *Chamobates cuspidatus* (Michael, 1884); Cham_subg, *Chamobates subglobulus* (Oudemans, 1900); Cham_voig, *Chamobates voigtsi* (Oudemans, 1902); Dama_ripa, *Damaeus riparius* Nicolet, 1855; Diss_orna, *Dissorhina ornata* (Oudemans, 1900); Enio_minu, *Eniochthonius minutissimus* (Berlese, 1903); Eulo_riba, *Eulohmannia ribagai* (Berlese, 1910); Eupe_hirt, *Eupelops hirtus* (Berlese, 1916); Eupe_plic, *Eupelops plicatus* (C.L. Koch, 1835); Galu_lanc, *Galumna lanceata* (Oudemans, 1900); Hypo_lute, *Hypochthonius luteus* Oudemans, 1917; Hypo_rufu, *Hypochthonius rufulus* C.L. Koch, 1835; Meta_pulv, *Metabelba pulverosa* Strenzke, 1953; Micr_mini, *Microtritia minima* (Berlese, 1904); Micr_minu, *Microppia minus* (Paoli, 1908); Nanh_nana, *Nanhermannia nana* (Nicolet, 1855); Noth_silv, *Nothrus sylvestris* Nicolet, 1855; Oppi_falc, *Oppiella falcata* (Paoli, 1908); Oppi_nova, *Oppiella nova* (Oudemans, 1902); Oppi_obso, *Oppiella obsoleta* (Paoli, 1908); Oppi_prop, *Oppiella propinqua* Mahunka & Mahunka-Papp, 2000; Oppi_subp, *Oppiella subpectinata* (Oudemans, 1900); Orib_calc, *Oribatella calcarata* (C.L. Koch, 1835); Orib_tibi, *Oribatula tibialis* (Nicolet, 1855); Pant_paol, *Pantelozetes paolii* (Oudemans, 1913); Phth_comp, *Phthiracarus compressus* Jacot, 1930; Phth_glob, *Phthiracarus globosus* (C.L. Koch, 1841); Phth_laev, *Phthiracarus laevigatus* (C.L. Koch, 1844); Phth_lent, *Phthiracarus lentulus* (C.L. Koch, 1841); Phth_long, *Phthiracarus longulus* (C.L. Koch, 1841); Plat_pelt, *Platynothrus peltifer* (C.L. Koch, 1839); Poro_spin, *Porobelba spinosa* (Sellnick, 1920); Quad_hamm, *Quadroppia hammerae* Minguez, Ruiz & Subias, 1985); Quad_mons, *Quadroppia monstruosa* Hammer, 1979 (sensu Minguez, Ruiz & Subias 1985); Quad_quad, *Quadroppia quadricarinata* (Michael, 1885); Rhys_dupl, *Rhysotritia duplicata* (Grandjean, 1953); Sche_init, *Scheloribates initialis* (Berlese, 1908); Steg_herc, *Steganacarus herculeanus* Willmann, 1953; Steg_magn, *Steganacarus magnus* (Nicolet, 1855); Sucto_sp., Suctobelbidae; Tect_alat, *Tectocepheus velatus alatus* Berlese, 1913; Tect_mino, *Tectocepheus minor* Berlese, 1903; Tect_sare, *Tectocepheus velatus sarekensis* Trägardh, 1910; Tect_vela, *Tectocepheus velatus velatus* (Michael, 1880).
